# Perlecan, a heparan sulfate proteoglycan, regulates systemic metabolism with dynamic changes in adipose tissue and skeletal muscle

**DOI:** 10.1038/s41598-018-25635-x

**Published:** 2018-05-17

**Authors:** Yuri Yamashita, Satoshi Nakada, Toshinori Yoshihara, Takeshi Nara, Norihiko Furuya, Takashi Miida, Nobutaka Hattori, Eri Arikawa-Hirasawa

**Affiliations:** 1 Aging Biology in Health and Disease, Juntendo University Graduate School of Medicine, Tokyo, 113–8421 Japan; 2 Department of Neurology, Juntendo University Graduate School of Medicine, Tokyo, 113–8421 Japan; 3 Japanese Center for Research on Women in Sport, Juntendo University Graduate School of Health and Sports Science, Chiba, 270–1695 Japan; 4 Department of Exercise Physiology, Juntendo University Graduate School of Health and Sports Science, Chiba, 270–1695 Japan; 5 Faculty of Pharmacy, Iwaki Meisei University, Fukushima, 970–8551 Japan; 6 Department of Clinical Laboratory medicine, Juntendo University Graduate School of Medicine, Tokyo, 113–8421 Japan; 7 Research Institute for Disease of Old Age, Juntendo University Graduate School of Medicine, Tokyo, 113–8421 Japan

## Abstract

Perlecan (HSPG2), a heparan sulfate proteoglycan, is a component of basement membranes and participates in a variety of biological activities. Here, we show physiological roles of perlecan in both obesity and the onset of metabolic syndrome. The perinatal lethality-rescued perlecan knockout (*Hspg2*^−/−^-Tg) mice showed a smaller mass and cell size of white adipose tissues than control (WT-Tg) mice. Abnormal lipid deposition, such as fatty liver, was not detected in the *Hspg2*^−/−^-Tg mice, and those mice also consumed more fat as an energy source, likely due to their activated fatty acid oxidation. In addition, the *Hspg2*^−/−^-Tg mice demonstrated increased insulin sensitivity. Molecular analysis revealed the significantly relatively increased amount of the muscle fiber type IIA (X) isoform and a larger quantity of mitochondria in the skeletal muscle of *Hspg2*^−/−^-Tg mice. Furthermore, the perlecan-deficient skeletal muscle also had elevated levels of peroxisome proliferator-activated receptor gamma coactivator 1-alpha (PGC1α) protein. PGC1α expression is activated by exercise, and induces mitochondrial biosynthesis. Thus, perlecan may act as a mechano-regulator of catabolism of both lipids and glucose by shifting the muscle fiber composition to oxidative fibers. Our data suggest that downregulation of perlecan is a promising strategy to control metabolic syndrome.

## Introduction

In recent years, human lifestyles have been largely changed, and metabolic syndrome is now threatening human health by enhancing the risks of cardiovascular disease and diabetes mellitus^1–3^. Metabolic syndrome is characterized by symptoms, such as visceral obesity, glucose intolerance, dyslipidemia, and hypertension, which are all related to each other and caused by metabolic disturbance. Adipose tissue and skeletal muscle are representative organs for the regulation of systemic metabolic dynamics, and both show dynamic changes in morphology and functions in response to environmental stimuli, such as diet and exercise conditions.

Adipose tissue acts as a fat storage depot in terms of energy sources, and it protects against abnormal lipid deposition in other organs^4^. Adipose tissue is also well recognized as the largest endocrine organ^5^; that is, adipocytes secrete a variety of adipocytokines. Smaller sized adipocytes contribute to the prevention of metabolic syndrome by secreting a unique cytokine, adiponectin, which elicits anti-inflammatory effects and enhances insulin sensitivity. By contrast, hypertrophic adipocytes degrade the metabolic status by the secretion of different type of cytokines, such as tumor necrosis factor-alpha (TNF-α), interleukin-6 (IL-6), and resistin^6^. The development of metabolic syndrome is therefore associated with dynamic remodeling of both adipose tissue and adipocytes^7,8^.

Skeletal muscle is also a dynamic metabolic organ, with four classifications based on the muscle fiber type. Types I, IIA, IIX, and IIB are distinguished by the maximum speed of contraction and the attributed energy metabolism^9,10^. Type I is a slow-twitch fiber and relies on oxidative phosphorylation. Type IIA is fast and oxidative while type IIX is fast, oxidative, and glycolytic. Type IIB is fast and glycolytic. The maximum speed of muscle contraction varies among the type II fibers with IIB as the fastest and IIX faster than IIA^11,12^. In response to exercise, the mass and composition of muscle fibers can change^13^.

Importantly, extracellular matrices (ECMs) modify dynamically their own structure and repertoires to affect metabolic environments^14^. Remodeling of ECMs is important for the reconstruction of tissue architecture during development, wound healing, pathological processes, etc^15^. ECMs regulate the functional as well as the structural microenvironment by interaction with various humoral ligands, such as growth factors^16,17^. The dynamics of ECMs are therefore associated with increased insulin resistance in skeletal muscle^18^, and with differentiation and remodeling of adipocytes in response to energy balance^19,20^.

Perlecan, a heparan sulfate proteoglycan, is one of components of ECMs. Perlecan contains 5 domains and can interact with basement membranes, growth factors, cell surface receptors, etc^21^. Perlecan surrounds individual adipocytes and skeletal muscle fibers, and therefore plays critical roles in the maintenance of the morphology and functions of adipose tissue and skeletal muscle. In addition, the heparan sulfate chains in domain I^22^ and domain II^23^ of perlecan bind to lipoproteins. Therefore, perlecan has been implicated in the regulation of the lipid dynamics of adipose tissue.

Our previous study showed that perlecan deficiency in plantaris muscle, a fast-twitch muscle, promotes hypertrophy under mechanical overload. This structural change is accompanied by reduced levels of myostatin, a negative regulator of muscle growth and differentiation, expression and signaling^24^. On the other hand, mechanical unloading on the soleus muscle, a slow-twitch muscle, induces a more substantial atrophy in perinatal lethality-rescued perlecan knockout (*Hspg2*^−/−^-Tg) mice than that in control mice, and this is accompanied by increased autophagic activity^25^. Consequently, perlecan may play a key role in the regulation of the dynamics of skeletal muscle in response to mechanical stimuli.

In the present study, we investigated the physiological roles of perlecan in both adipose tissue and skeletal muscle in terms of the regulation of metabolic status. Our results established the metabolic linkages between perlecan and both adipose tissue and skeletal muscle are discussed.

## Results

### *Hspg2*^−/−^-Tg mice are resistant to obesity

**Figure 1:**
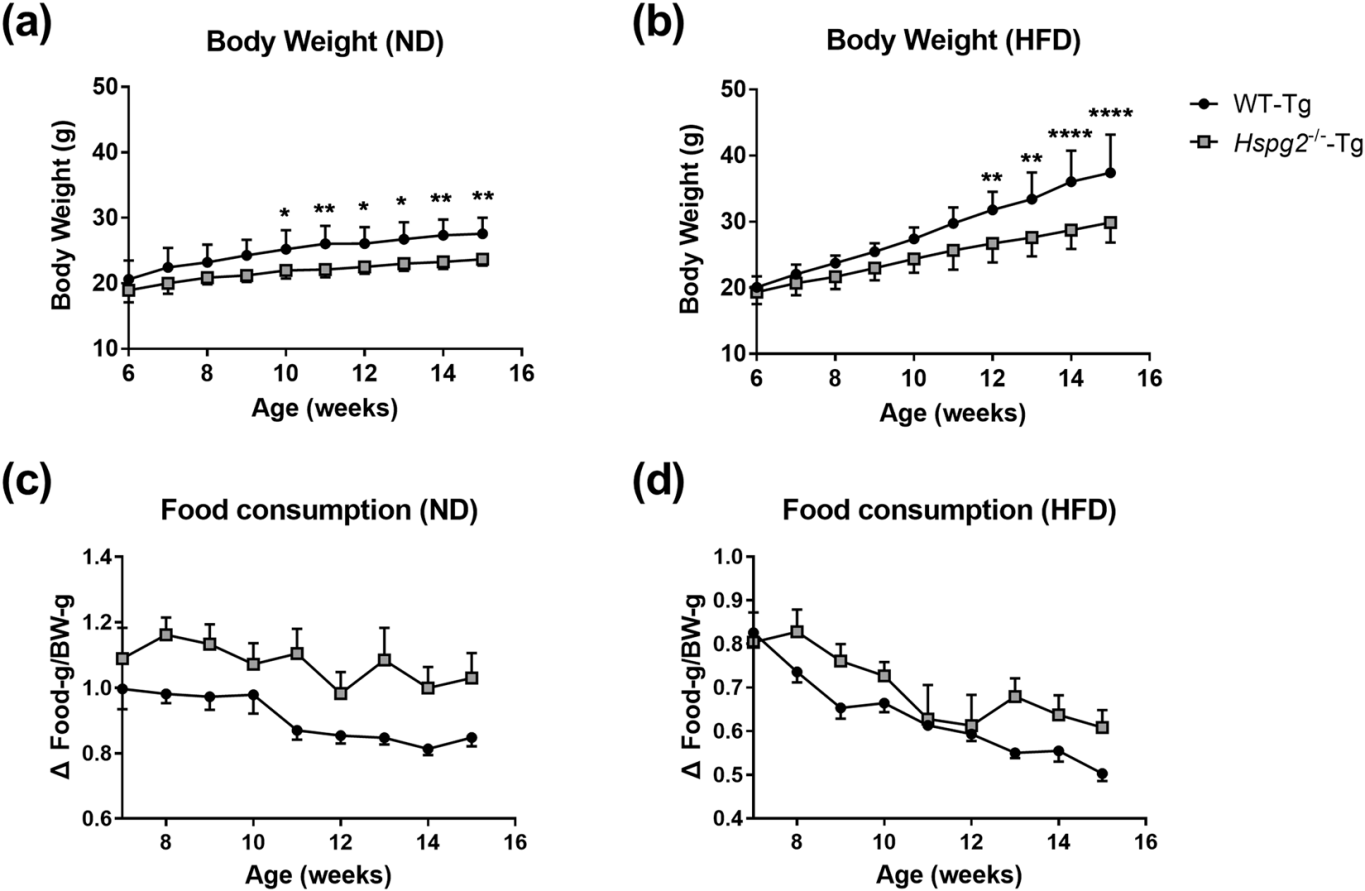
*Hspg2*^−/−^-Tg mice are resistant to obesity. (**a**,**b**) Body weight change of perinatal lethality-rescued perlecan knockout (*Hspg2*^−/−^-Tg, gray square) and control (WT-Tg, black circle) mice under (**a**) normal diet (ND) and (**b**) high fat diet (HFD) conditions. Body weight was monitored in the mice aged 6 to 16 weeks (mean ± S.D., n = 7). (**c**,**d**) Food consumption by WT-Tg and *Hspg2*^−/−^-Tg mice under (**c**) ND and (**d**) HFD conditions. The average consumption by 1 to 3 mice reared in the same animal cage represents food consumption per mouse (mean ± S.D., n = 4–6 cages). Data were analyzed by two-way ANOVA with Sidak’s multiple comparison. *p < 0.05, **p < 0.01, ****p < 0.0001.

We investigated the physiological relationships between perlecan and obesity using *Hspg2*^−/−^-Tg mice. The mice were fed either a normal diet (ND) or a high fat diet (HFD) from 6 to 16 weeks of age. The relevant macroscopic features between *Hspg2*^−/−^-Tg and the background (WT-Tg) mice were evaluated. By 6 weeks of age, the body weight did not differ significantly between *Hspg2*^−/−^-Tg and WT-Tg mice. After 6 weeks of age, the age-dependent weight gain of the *Hspg2*^−/−^-Tg mice was lower than that of the WT-Tg mice in both nutritional conditions. These trends became significant after 10 weeks of age in the ND (*p* = 0.0420, at 10 weeks of age; *p* = 0.0068, at 15 weeks of age; Fig. 1a), and after 12 weeks of age in the HFD conditions (*p* = 0.0086, at 12 weeks of age; *p* < 0.0001, at 15 weeks of age; Fig. 1b). The *Hspg2*^−/−^-Tg mice did not show reduced levels of food consumption when compared to the WT-Tg mice in either nutritional condition (Fig. 1c,d).

**Figure 2:**
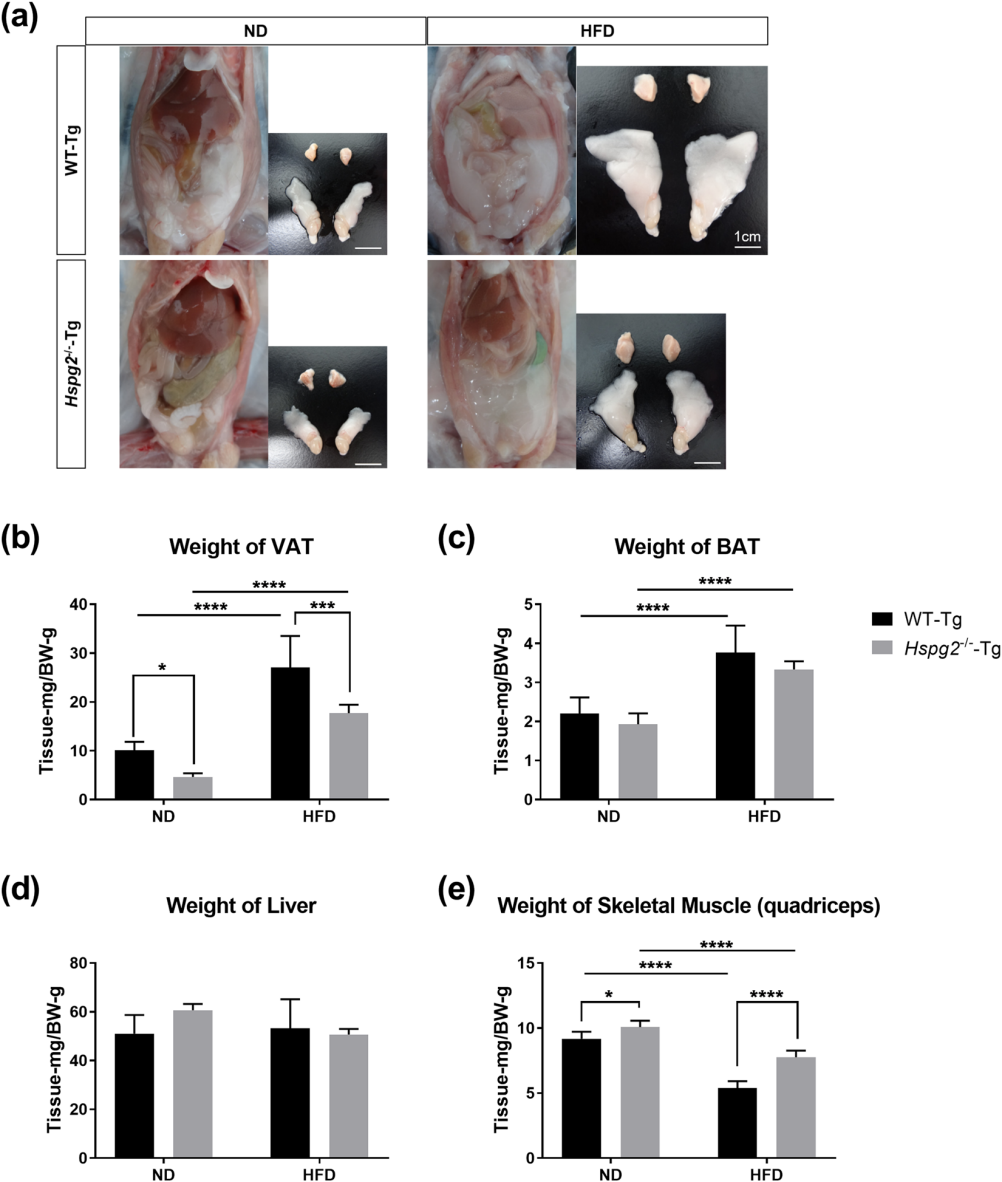
The mass of white adipose tissue is reduced in *Hspg2*^−/−^-Tg mice. (**a**) Macroscopic images of visceral fat deposition and adipose tissues of the WT-Tg (upper panels) and the *Hspg2*^−/−^-Tg (lower panels) mice at 16 weeks of age. The right-hand image of each panel represents brown adipose tissue (interscapular fats, upper row) and visceral adipose tissue (epididymal fats, bottom row). (**b–e**) Comparisons of tissue weight of (**b**) visceral adipose tissue (VAT), (**c**) brown adipose tissue (BAT), (**d**) liver, and (**e**) skeletal muscle (quadriceps) in mice at 16 weeks of age fed the different diets. Data points and error bars represent the mean ± S.D. (n = 7). Data were analyzed by two-way ANOVA with Tukey’s multiple comparison. *p < 0.05, ***p < 0.001, ****p < 0.0001. Scale bar, 1 cm.

We also compared the mass of adipose tissue in WT-Tg and *Hspg2*^−/−^-Tg mice. Macroscopically, the mass of epididymal adipose tissue, a representative visceral white adipose tissue (VAT), was smaller in the *Hspg2*^−/−^-Tg mice than in the WT-Tg mice under the ND condition. The VAT became enlarged in both genotypes under the HFD condition; however, this enlargement was more prominent in the WT-Tg than in the *Hspg2*^−/−^-Tg mice (Fig. 2a). The relative weight of the VAT per body was significantly reduced in the *Hspg2*^−/−^-Tg mice, irrespective of the nutritional conditions (*p* = 0.0369, in ND; *p* = 0.0002, in HFD; Fig. 2b). By contrast, we did not observe such differences in brown adipose tissue (BAT) or in the liver between genotypes, whereas HFD promoted an increase in BAT mass in both animal types (Fig. 2c,d). In the skeletal muscle, the *Hspg2*^−/−^-Tg mice showed a significant increase in weight under both nutritional conditions (*p* = 0.0173, in ND; *p* < 0.0001, in HFD; Fig. 2e). These results suggest that perlecan deficiency may not lead to systemic hypoplasia, but instead may promote a reduction in the fat storage of white adipose tissue, and thereby prevent obesity.

### *Hspg2*^−/–^-Tg mice show reduced size of white adipocytes

**Figure 3:**
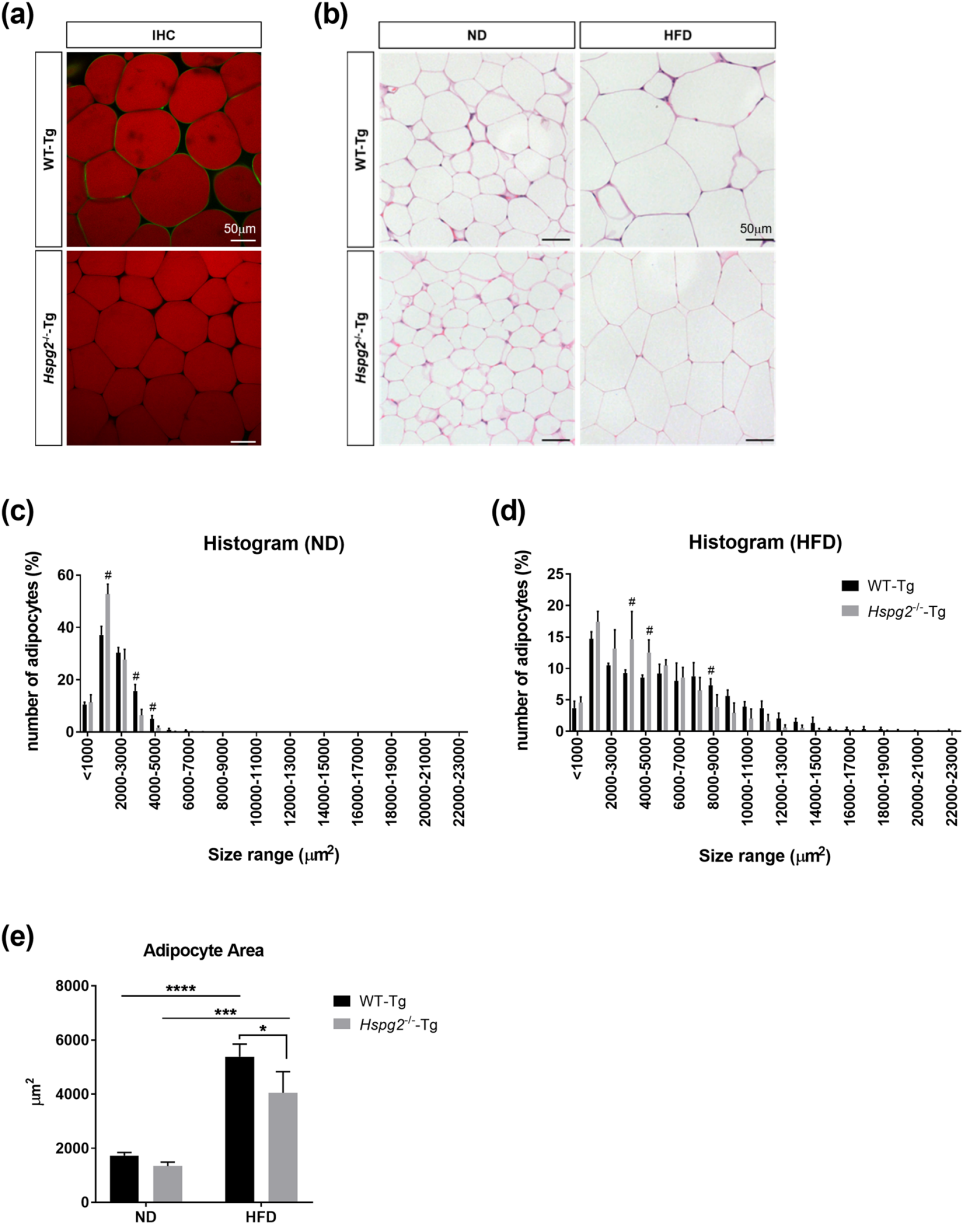
Perlecan deficiency leads to a reduction in adipocyte size. (**a**) Immunofluorescence analysis (IFA) of perlecan in visceral adipose tissue (VAT). Note that perlecan (green) surrounded each adipocyte (red) of the WT-Tg mice, whereas perlecan was absent in that of the *Hspg2*^−/−^-Tg mice. (**b**) The VAT of the WT-Tg (upper panel) and *Hspg2*^−/−^-Tg (lower panel) mice fed with either a normal diet (ND) or a high-fat diet (HFD). A section was stained with hematoxylin-eosin. (**c**,**d**) A histogram of the size (μm^2^) of the individual adipocytes under (**c**) ND and (**d**) HFD conditions. (**e**) The average size of adipocytes (μm^2^). (**c–e**) 1,000 adipocytes per mouse were evaluated. Data points and error bars represent the mean ± S.D. (n = 3–4). Data were analyzed by two-way ANOVA with Sidak’s multiple comparison (**c**,**d**) and Tukey’s multiple comparison (**e**). ^#^*p* < 0.05 including *p* < 0.0001, *p < 0.05, ***p < 0.001, ****p < 0.0001. Scale bar, 50  μm.

We performed a microscopy analysis to investigate the size of adipocytes in *Hspg2*^−/−^-Tg mice. We first confirmed the localized distribution of perlecan in adipose tissue. Immunofluorescence analysis revealed that perlecan surrounds each adipocyte in the VAT of WT-Tg mice, whereas perlecan was absent in the VAT of *Hspg2*^−/−^-Tg mice (Fig. 3a). Hematoxylin and eosin staining of the VAT demonstrated a reduced cell size of the white adipocytes in the *Hspg2*^−/−^-Tg mice under both nutritional conditions (Fig. 3b). Histograms of adipocyte size also revealed an increased number of smaller cells in the *Hspg2*^−/−^-Tg mice (*p* < 0.0001, in 1,000–2,000 μm^2^), associated with an decreased number of larger cells (*p* < 0.0001, in 3,000–4,000 μm^2^) in the ND condition (Fig. 3c). Such a trend was also observed in the HFD condition (*p* < 0.0001 in 3,000–4,000 μm^2^; *p* = 0.0345 in 8,000–9,000 μm^2^; Fig. 3d). Figure 3e shows the average size of the adipocytes; these cells were significantly smaller in the *Hspg2*^−/−^-Tg mice in the HFD condition (*p* = 0.0206), whereas no significant difference was observed in the ND condition.

**Figure 4:**
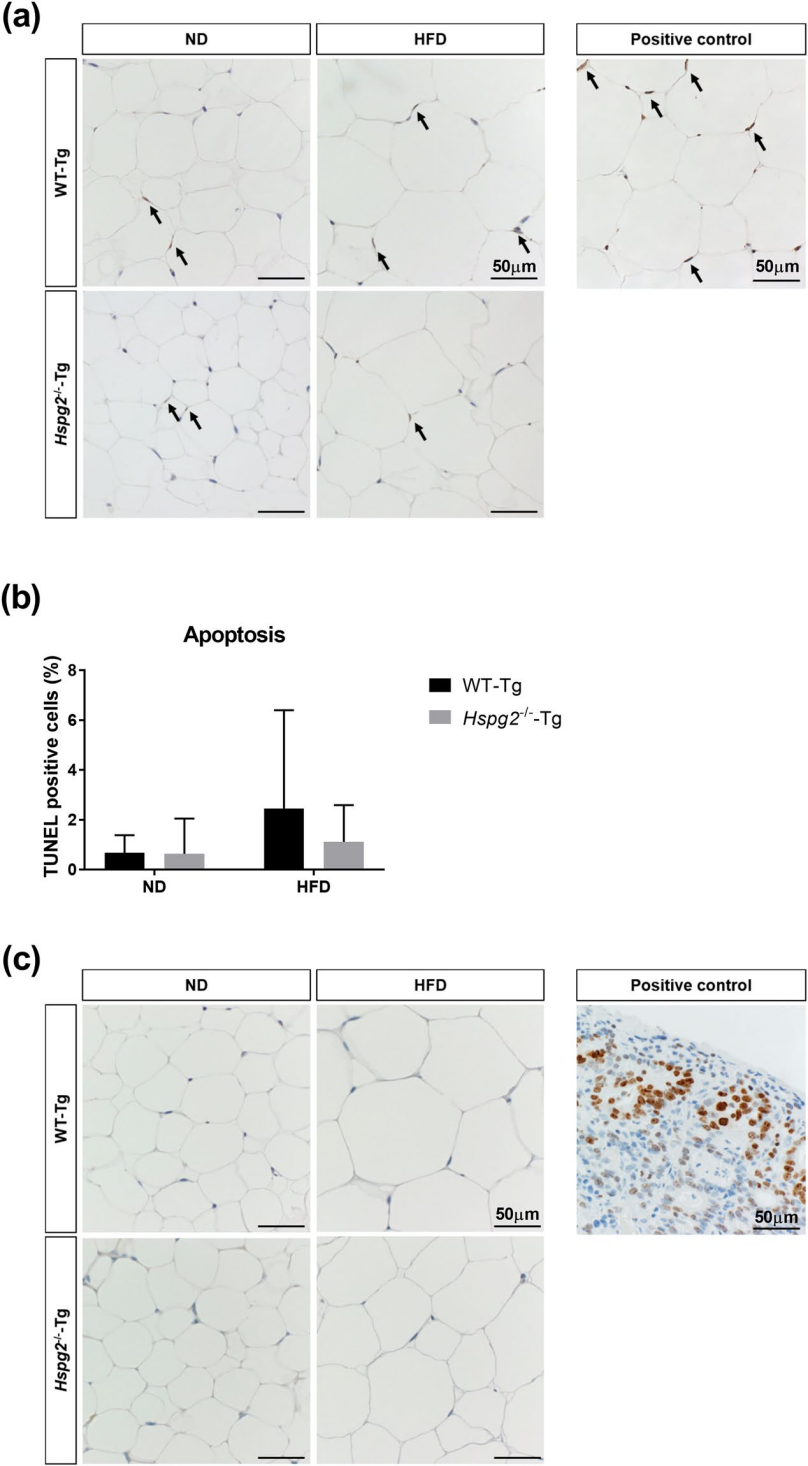
Perlecan deficiency does not affect cell turnover in white adipose tissue. (**a**) TUNEL staining in the VAT of the WT-Tg (upper panel) and the *Hspg2*^−/−^-Tg (lower panel) mice fed either ND or HFD. The areas containing positive nuclei of adipocytes are selected and shown. The positive control was made using DNase I. Note that TUNEL-positive nuclei are brown and negative nuclei are blue. (**b**) The percentage of TUNEL-positive nuclei of adipocytes. The nuclei from at least 100 adipocytes per mouse were evaluated. Data points and error bars represent the mean ± S.D. (n = 5). (**c**) Representative immunohistochemical staining of Ki67 in the VAT of the WT-Tg (upper panel) and the *Hspg2*^−/−^-Tg (lower panel) mice fed either ND or HFD. The tissue from human abdominal cancer was used as positive control. No nuclei were positive for Ki67 in any groups. Data were analyzed by two-way ANOVA with Tukey’s multiple comparison (**b**). Scale bar, 50  μm.

We also compared the turnover of adipocytes in the *Hspg2*^−/−^-Tg mice with that in the WT-Tg mice to investigate whether the smaller VAT mass in the perlecan deficient mice is due to either increased apoptosis or decreased proliferation of adipocytes. TUNEL assays of apoptotic cells demonstrated that the number of apoptotic adipocytes was not significantly higher in the *Hspg2*^−/−^-Tg mice than in the WT-Tg mice (Fig. 4a,b). We could not detect the protein expression of Ki67, a marker of cell proliferation, in the adipose tissue of either the *Hspg2*^−/−^-Tg or the WT-Tg mice (Fig. 4c).

These observations suggest that the loss of perlecan inhibits lipid accumulation in individual adipocytes, but it does not affect cell turnover in white adipose tissue.

### Perlecan deficiency does not induce abnormal lipid deposition

**Figure 5:**
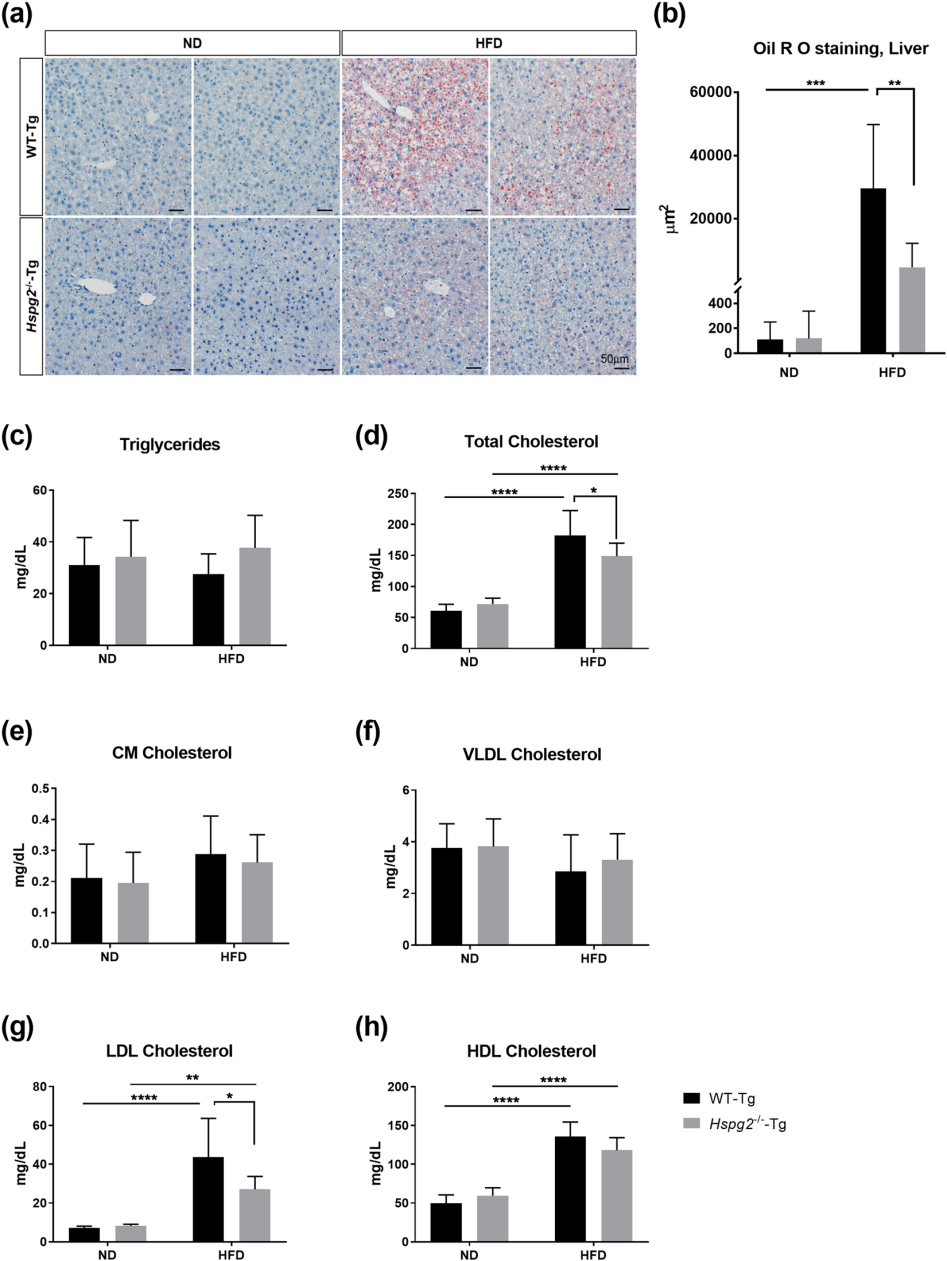
Perlecan deficiency is resistant to abnormal lipid deposition. (**a**) Histological analysis of lipid deposition in the livers of the WT-Tg (upper panel) and *Hspg2*^−/−^-Tg (lower panel) mice fed either a normal diet (ND) or a high fat diet (HFD). A section was stained with Oil Red O for detection of lipids and with hematoxylin for counterstaining. Two representative images are shown for each experimental condition. (**b**) The differences in the lipid deposition between the WT-Tg and the *Hspg2*^−/−^-Tg mice fed the ND and HFD. Areas (μm^2^) of deposited lipids per image with same magnification were calculated using ImageJ software. Sixteen-week-old mice were used in the experiments. Data points and error bars represent the mean ± S.D. (n = 7). (**c**–**h**) Levels (mg/dL) of plasma (**c**) triglycerides, (**d**) total cholesterol, (**e**) chylomicron (CM) cholesterol, (**f**) very low density lipoprotein (VLDL) cholesterol, (**g**) low density lipoprotein (LDL) cholesterol, and (**h**) high density lipoprotein (HDL) cholesterol in the 16-week-old mice after fasting for 4 h (mean ± S.D., n = 8). Data were analyzed by two-way ANOVA with Tukey’s multiple comparison. *p < 0.05, **p < 0.01, ***p < 0.001, ****p < 0.0001. Scale bar, 50  μm.

Adipose tissue plays an important role in the prevention of abnormal lipid deposition in other organs. Therefore, reduced fat storage in white adipose tissue could possibly reflect an enhancement of abnormal lipid deposition or dyslipidemia. We investigated whether abnormal lipid deposition is present in *Hspg2*^−/−^-Tg mice. Our histological analysis did not show any significant lipid deposition in the liver of *Hspg*2^−/−^-Tg mice in the ND condition, as in the liver of WT-Tg mice fed the ND. Even under the HFD condition, no significant fatty liver formation was observed in the *Hspg*2^−/−^-Tg mice, whereas the WT-Tg mice fed the HFD showed a high prominence of fatty liver (29,576 ± 18,714 μm^2^ in WT-Tg; 4,645 ± 7,033 μm^2^ in *Hspg*2^−/−^-Tg, *p* = 0.0013; Fig. 5a,b).

The plasma levels of triglycerides were not significantly elevated in the *Hspg2*^−/−^-Tg mice, even in the HFD condition (Fig. 5c). Notably, the plasma levels of total cholesterol were significantly lower in the *Hspg2*^−/−^-Tg mice than in the WT-Tg mice under the HFD condition (*p* < 0.0001, Fig. 5d). We also examined cholesterol concentrations in four major lipoproteins: chylomicrons (CM), very low density lipoproteins (VLDL), low density lipoproteins (LDL), and high density lipoproteins (HDL). The plasma level of LDL cholesterol was significantly lower in the *Hspg2*^−/−^-Tg mice than in the WT-Tg mice under the HFD condition (*p* = 0.0198, Fig. 5g), whereas the CM cholesterol, VLDL cholesterol, and HDL cholesterol levels were not significantly different between the genotypes under either nutritional condition (Fig. 5e,f,h). The CM are synthesized in the intestine from dietary triglycerides, and VLDL are synthesized in the liver from endogenous triglycerides^26^. We conclude that an enhanced abnormal lipid deposition is unlikely, and that the fat storage alone is reduced in *Hspg2*^−/−^-Tg mice. Furthermore, this reduction is not caused by suppression of lipid synthesis in the intestine and liver.

### Fatty acid oxidation is elevated in *Hspg2*^−/−^-Tg mice

**Figure 6:**
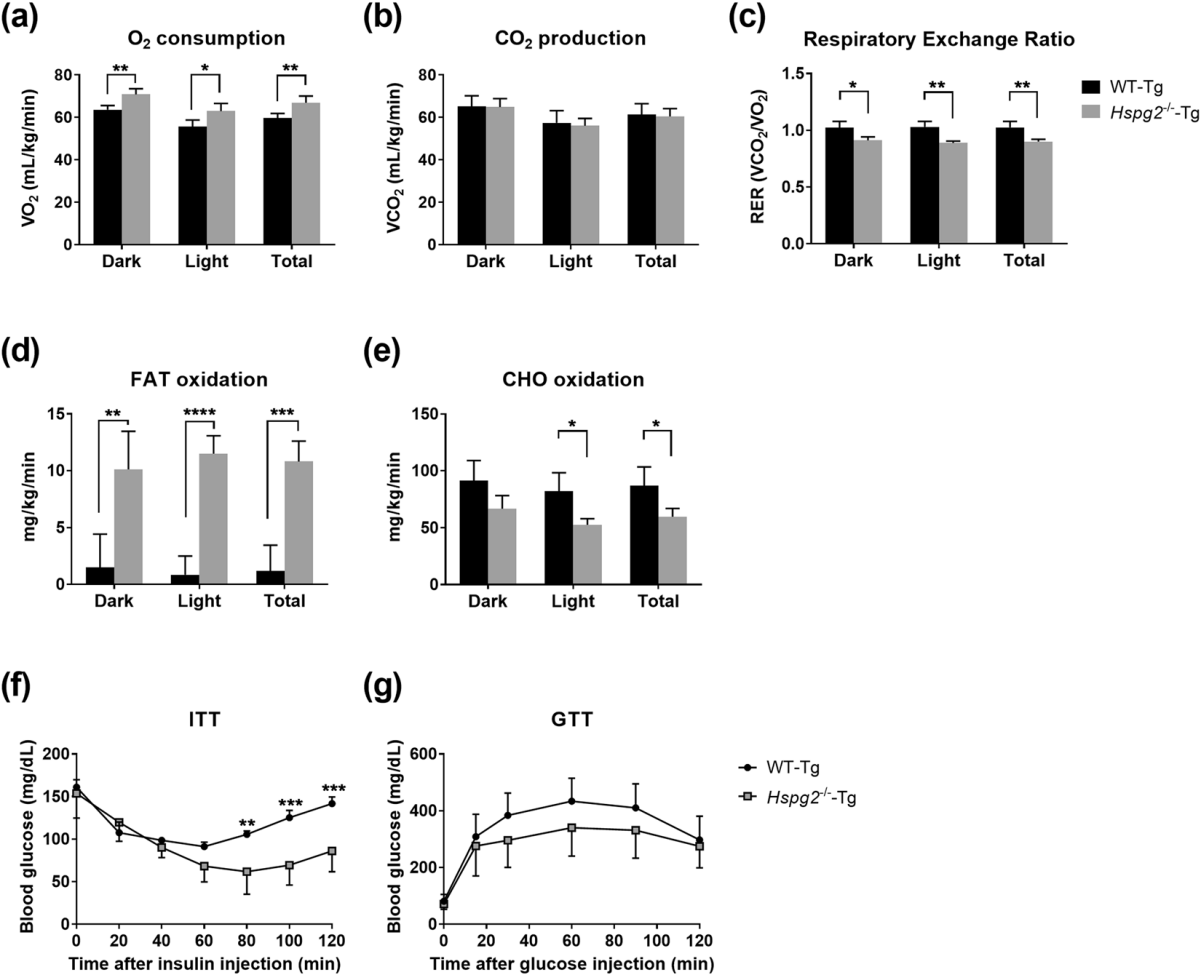
Anti-obesity effects in *Hspg2*^−/−^-Tg mice depend on increased FAT oxidation. (**a**–**e**) Indirect calorimetry for (**a**) oxygen (O_2_) consumption, (**b**) CO_2_ production, (**c**) respiratory exchange ratio, (**d**) FAT oxidation, and (**e**) carbohydrate (CHO) oxidation. Data were collected every 2.5 min for 24 h and represent the mean ± S.D. (n = 4) during 6 h light/6 h dark periods. (**f**,**g**) Changes in plasma glucose levels by (**f**) the insulin tolerance test (ITT) and (**g**) the glucose tolerance test (GTT). Data points and error bars represent the mean ± S.D. (n = 4–7). Data were analyzed by the unpaired *t*-test (**a**–**e**) and two-way ANOVA with Sidak’s multiple comparison (**f**,**g**). *p < 0.05, **p < 0.01, ***p < 0.001, ****p < 0.0001.

Lipid accumulation in the white adipose tissue and liver was suppressed in the *Hspg2*^−/−^-Tg mice, as was dyslipidemia. By contrast, neither food consumption nor lipid synthesis in the intestine and liver were suppressed in these mice. Our findings raised the possibility that the reduction in lipid storage in the *Hspg2*^−/−^-Tg mice was due to an acceleration of lipid catabolism. Therefore, we investigated lipid and glucose metabolism in the *Hspg2*^−/−^-Tg mice fed ND. We found a significantly increased whole-body oxygen (O_2_) consumption in the *Hspg2*^−/−^-Tg mice (dark period, *p* = 0.0047; light period, *p* = 0.0218; total period, *p* = 0.0092; Fig. 6a), whereas CO_2_ production did not differ significantly between the two genotypes (Fig. 6b). Colorimetric analysis further demonstrated a lower respiratory exchange ratio (RER) in the *Hspg2*^−/−^-Tg than in the WT-Tg mice (dark period, *p* = 0.0126; light period, *p* = 0.0015; total period, *p* = 0.0040; Fig. 6c), which indicated significantly higher levels of fat oxidation in the *Hspg2*^−/−^-Tg than in the WT-Tg mice (dark period, *p* = 0.0084; light period, *p* < 0.0001; total period, *p* = 0.0006; Fig. 6d). By contrast, carbohydrate (CHO) oxidation levels were significantly lower in the *Hspg2*^−/−^-Tg mice during the light and the total period (light period, *p* = 0.0146; total period, *p* = 0.0256; Fig. 6e). Notably, the insulin tolerance test (ITT) demonstrated a greater sensitivity to insulin in the *Hspg2*^−/−^-Tg than in the WT-Tg mice; that is, blood glucose levels were significantly lower in the *Hspg2*^−/−^-Tg mice (*p* = 0.0084, at 80 min after insulin injection, Fig. 6f), whereas the glucose tolerance test (GTT) did not show a significant difference between the genotypes (Fig. 6g). These data suggest that fats are the more preferred energy source in *Hspg2*^−/−^-Tg mice without induced insulin resistance. We conclude that the increased fat oxidation may contribute to the resistance to obesity in *Hspg2*^−/−^-Tg mice.

### Loss of perlecan causes muscle fiber shift from glycolytic to oxidative fibers by activation of PGC1α

Skeletal muscle has a physiological role in regulating fatty acid oxidation and insulin sensitivity. Indeed, exercise enhances fatty acid oxidation and prevents insulin resistance in skeletal muscle^27^. We previously found that perlecan regulates skeletal muscle dynamics, so we hypothesized that alterations in skeletal muscle by perlecan deficiency would increase β-oxidation and insulin sensitivity. β-oxidation occurs more prominently in oxidative skeletal muscles, in which mitochondria are enriched. Therefore, we investigated the differences in muscle fiber composition between *Hspg2*^−/−^-Tg mice and WT-Tg mice. We used the quadriceps as a representative skeletal muscle, because it is the largest skeletal muscle in the mouse body^28^.

**Figure 7:**
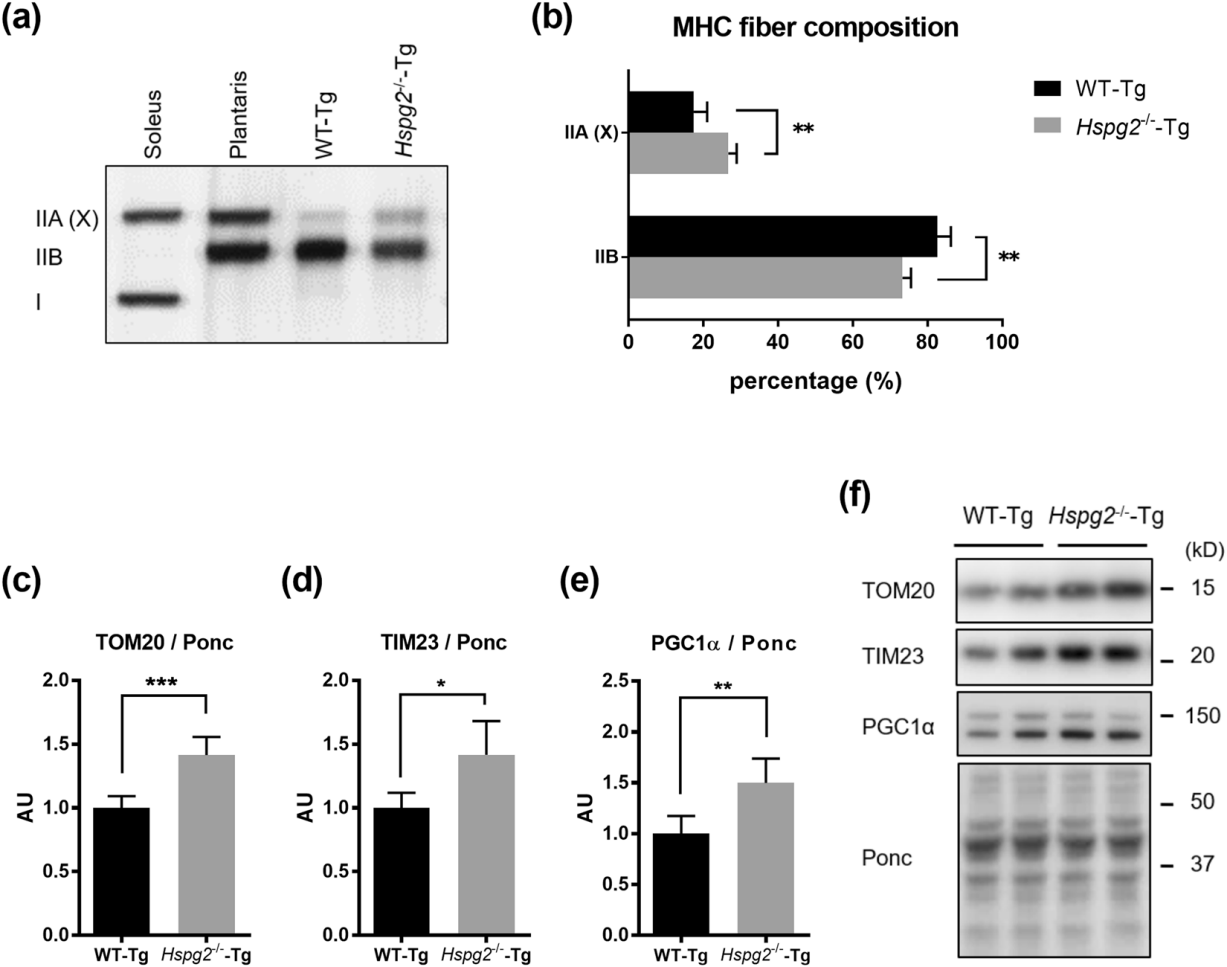
Loss of perlecan modifies the composition of myosin heavy chains by activating PGC1α. Detection by (**a**) SDS-PAGE-coupled silver staining and (**b**) relative composition of myosin heavy chain isoforms in the quadriceps of the WT-Tg and *Hspg2*^−/−^-Tg mice. Soleus and plantaris represent markers for type I and II fibers, respectively. The relative intensity of the bands was quantified using ImageJ software. (**c–e**) Protein expression levels of (**c**) translocase of outer membrane 20 (TOM20), (**d**) translocase of inner membrane 23 (TIM23), and (**e**) peroxisome proliferator-activated receptor gamma coactivator 1-alpha (PGC1α) in the quadriceps of the WT-Tg and *Hspg2*^−/−^-Tg mice. (**f**) Representative images of proteins extracted from quadriceps and stained with Ponceau S (Ponc) after SDS-PAGE. The relative intensities of the respective bands detected by western blotting using the specific antibody to the Ponc-stained patterns were quantified using ImageJ software. Data points and error bars represent the mean ± S.D. (n = 5 in **a** and **b**; n = 5–6 in **c**–**e**). Data were analyzed by two-way ANOVA with Sidak’s multiple comparison (**b**) and unpaired *t-*test (**c**–**e**). *p < 0.05, **p < 0.01, ***p < 0.001.

Myosin heavy chain (MHC) isoforms (type I, IIA, IIX, and IIB) can be distinguished by gel electrophoresis. The soleus and plantaris muscles are markers for type I and II fibers. In the soleus muscle, a slow-twitch muscle, type I fibers are dominant, whereas type II fibers predominate in the plantaris muscle. The MHC isoforms in the quadriceps showed a significant increase in the relative amounts of muscle fiber type IIA (X) in the *Hspg2*^−/−^-Tg mice (*p* = 0.0011; Fig. 7a, b), whereas the differences in type IIA from IIX fibers was variable as previously shown^29^. In addition, the levels of mitochondrial proteins, translocase of outer membrane 20 (TOM20) and translocase of inner membrane 23 (TIM23), were significantly increased in the perlecan-deficient muscle (*p* = 0.0003, in TOM20; Fig. 7c; *p* = 0.0104, in TIM23; Fig. 7d). These data indicated that perlecan-deficient muscle utilizes more oxygen than that of WT-Tg mice.

Peroxisome proliferator-activated receptor gamma coactivator 1-alpha (PGC1α), a transcriptional coactivator involved in energy metabolism, induces mitochondrial biogenesis and promotes the switching of muscle fiber types from glycolytic to more oxidative^30^. Our investigation of the expression level of PGC1α, as expected, revealed increased protein levels of PGC1α in the *Hspg2*^−/−^-Tg mice (*p* = 0.0038; Fig. 7e). Thus, the loss of perlecan causes dynamic changes in lipid metabolism by shifting the muscle fiber composition to more oxidative fibers via PGC1α.

## Discussion

Perlecan is a multi-functional heparan sulfate proteoglycan that interacts with a number of different molecules, including basement membrane components (e.g., laminin^31^, nidogen^32^, and collagen IV^33^), cell surface receptors (e.g., integrin^34,35^), growth factors (e.g., fibroblast growth factor (FGF) 2, FGF7, FGF18, FGF-BP, PDGF, and VEGF^36–38^), lipoproteins^23^, acetylcholinesterase^39^, and α-dystroglycan^40^.

Perlecan null mice are embryonic lethal due to chondrodysplasia; therefore, investigating the systemic physiological functions of perlecan is difficult. Several animal models have been created with mutations in *Hspg2*^21^. The *Hspg2*^Δ3/Δ3^ mice lacking exon 3 of the perlecan gene, which encodes for two of the three heparan sulfate attachment sites^41^, are particularly useful for investigating the function of the heparan sulfate chains. The perinatal lethality-rescued perlecan knockout (*Hspg2*^−/−^-Tg) mice that we have established express perlecan only in the cartilage, making them useful for investigating the function of the whole perlecan protein. The *Hspg2*^−/−^-Tg mice and WT-Tg mice display similar life expectancies, thereby allowing the study of the role of perlecan role throughout adulthood and in the aged state.

The heparan sulfate chains of perlecan play significant roles in lipid uptake and in the proliferation and differentiation of muscle cells through the association of these chains with lipoproteins^22,42^ and FGFs^43^. The FGFs promote muscle cell proliferation and inhibit cell differentiation^44^. Notably, previous studies have reported the importance of the perlecan core protein itself. Domain II, which is similar to the cholesterol binding region of the LDL receptor^23,45^, has a pro-atherosclerosis effect, and the C-terminal domain V, endorepelin, is a key regulator of muscle development^46^. We therefore used the *Hspg2*^−/−^-Tg mice to investigate the physiological roles of perlecan, including the core protein, in metabolic dynamics.

In the present study, the *Hspg2*^−/−^-Tg mice showed a reduction in white adipose tissue weight. The cell proliferation and apoptosis in the adipose tissue of the *Hspg2*^−/−^-Tg mice were similar to that of the WT-Tg mice. Conversely, the size of the adipocytes was significantly smaller in the perlecan-deficient mice than in the control. Our findings indicated that a deficiency of perlecan does not affect cell turnover in adipose tissue, but it does decrease lipid accumulation. We also found that lipid accumulation in the liver was also suppressed in the *Hspg2*^−/−^-Tg mice under the HFD condition. Cell surface heparan sulfate proteoglycans (HSPGs), such as syndecans and glypicans, bind to both lipoproteins and lipoprotein lipase through the heparan sulfate chains^47^. Hence, HSPGs promote lipid uptake into both adipocytes and hepatocytes^42,48^. Because perlecan contains the multiple responsible sites for heparan sulfate side chains; 3 sites in domain I and one in domain V, perlecan can also play an important role in lipid accumulation. Indeed, previous reports proposed that the perlecan heparan sulfate in domain I has suggested a proatherogenic effect due to lipoprotein binding^22^. In addition, a recent study has shown that the perlecan domain II induced atherosclerosis^23^. Thus, the reduced lipid accumulation in the perlecan-deficient *Hspg2*^−/−^-Tg mice would seem to confirm these roles for perlecan.

Conversely, our observations that plasma levels of total cholesterol and triglycerides in the *Hspg2*^−/−^-Tg mice did not increase even under the HFD condition seem controversial to the aforementioned findings. The plasma levels of CM cholesterol and VLDL cholesterol were not suppressed in the *Hspg2*^−/−^-Tg mice, indicating that the loss of perlecan does not affect lipid synthesis in the intestine or the liver. A possible explanation is that *Hspg2*^−/−^-Tg mice have a greater lipid catabolism than WT-Tg mice, and this hypothesis is supported by our data indicating an activation of β-oxidation in the *Hspg2*^−/−^-Tg mice. We evaluated the skeletal muscle in the *Hspg2*^−/−^-Tg mice, because this tissue is responsible for activating β-oxidation. Skeletal muscles from the *Hspg2*^−/−^-Tg mice showed a significant relevant increase in the type IIA(X)/IIB ratio, suggesting an activation of β-oxidation. In mouse muscle fibers, the SDH activity is highest in type IIA and lowest in IIB fibers^49^. The quantity of lipid droplets in muscle fibers, which are often found adjacent to mitochondria and are used as an energy source during exercise, is higher in both type IIA and IIX fibers, and lowest in type IIB fibers^50^. The increase in the relative amount of type IIA (X) fibers in perlecan-deficient muscle indicates that the loss of perlecan enhances oxidative metabolism. The significantly increased levels of mitochondrial proteins also support this hypothesis.

The *Hspg2*^−/−^-Tg mice also showed increased PGC1α protein levels. PGC1α expression is activated by exercise, and it induces mitochondrial biogenesis and ultimately activated oxidative metabolism^51–53^. Muscle-specific PGC1α-transgenic mice showed increased expression of transcripts for mitochondrial enzymes, such as COX II and COX IV^30^. Conversely, PGC1α deficiency in muscle caused fiber type shifting from oxidative to glycolytic muscle fibers^54^. Hence, elevation of PGC1α expression in type II fiber-rich muscle promotes fiber-type switching from glycolytic toward more oxidative fibers. In our previous study, perlecan-deficient plantaris muscle shows decreased levels of myostatin and increased levels of insulin-like growth factor (IGF)-1 as compared to WT-Tg^24^. Myostatin^55^ and IGF-1^56–58^ are important molecules in skeletal muscle function, and these expression levels are influenced by exercise, as well as PGC1α. Under mechanical loads, the skeletal muscles show decreased expression of myostatin^59^, but enhanced expression of IGF-1^60^. Taken together with these findings, the enhanced expression of PGC1α in perlecan-deficient skeletal muscle indicates that perlecan may act as a mechano-regulator. Furthermore, the loss of perlecan promotes the catabolism of both lipids and glucose by shifting the muscle fiber composition to oxidative fibers via PGC1α signaling.

## Methods

### Animals

Perinatal lethality-rescued perlecan knockout (*Hspg2*^−/−^-Tg) mice and the WT-Tg mice (control) were used in the study. Perlecan null (*Hspg2*^−/−^) mice are embryonic lethal due to dyschondroplasia^61,62^. We rescued the premature cartilage development using a chondrocyte-specific Col2a1 collagen promoter and enhancer^63^ to create a perlecan transgenic mouse line (WT-Tg, *Hspg2*^+/+^; *Col2a1*-*Hspg2*^Tg/−^). We subsequently established the lethality-rescued mice (*Hspg2*^−/−^-Tg, *Hspg2*^−/−^; *Col2a1-Hspg2*^*Tg*/−^) by mating the transgenic mice with heterozygous *Hspg2*^+/−^ mice^24^. We maintained these genetic backgrounds on C57BL/6 mice. Perlecan is absent from the skeletal muscle, liver, kidney, heart, and brain of the *Hspg2*^−/−^-Tg mice, whereas perlecan is expressed in all these tissues in the WT-Tg mice, and the recombinant perlecan is expressed in the cartilage of *Hspg2*^−/−^-Tg mice^24,64^. The mice were reared under a 12-h light-dark cycle at 23 ± 2 °C, housed in groups of 1–3 per cage, and fed a normal diet (CRF-1, ORIENTAL YEAST CO., LTD.). For the high fat diet, the animal food containing 60 kcal% fat (D12492, Research Diets, Inc.) was fed to the mice for 10 weeks when they were 6 to 16 weeks of age. Body weight and food consumption were monitored every week from 6 to 16 weeks of age. The average food consumption per cage containing 1–3 mice was used as food consumption per mouse (n = 4–6 cages). At 16 weeks old, the mice were sacrificed after fasting for 4 h and various metabolic tissues, including visceral white adipose tissue (VAT), brown adipose tissue (BAT), liver, and skeletal muscle were collected. The VAT and BAT were dissected from epididymal and scapular regions, respectively. The quadriceps was used as the skeletal muscle. All animal experiments of this study were carried out exclusively in accordance with the Fundamental Guidelines for Proper Conduct of Animal Experiment and Related Activities in Academic Research Institutions under the jurisdiction of the Ministry of Education, Culture, Sports, Science and Technology (Notice No. 71, 2006), and approved by the Committee for Animal Experimentation of Juntendo University with the Approval No. 290207. The present study did not include research involving human participants, as well as non-human primates and unpublished de novo cell lines.

### Immunofluorescence analysis (IFA)

Immunofluorescence analysis of perlecan and adipocytes was performed as described previously^65^. Briefly, the VAT was cut into small pieces (about 4 mm square) using scalpels. These pieces were washed in 1 × PBS for 10 min, fixed in 4% paraformaldehyde (PFA) for 45 min, and permeabilized with 1% Triton X-100 for 10 min. After washing with 1 × PBS for 10 min, the pieces were blocked with 1% bovine serum albumin (BSA) for 30 min and incubated overnight with rat anti-mouse HSPG monoclonal antibody (Clone A7L6, MAB1948P, Merck KGaA) at appropriate dilutions. The specimens were washed six times with 1 × PBS for 10 min, and then reacted with Alexa Fluor 546-labeled goat anti-rat IgG (A11081, Thermo Fisher Scientific Inc.). The whole mount was counterstained with 5 μM BODIPY^TM^ 493/503 (D-3922, Thermo Fisher Scientific Inc.) for visualizing adipocytes. Microscopy observations were carried out using a confocal fluorescence microscope.

### Evaluation of the adipocyte sizes in VAT

The adipocyte counts were evaluated by morphometric analysis using hematoxylin-eosin (HE) stained VAT sections. The VAT whole mount was fixed overnight with 4% PFA, embedded in paraffin, sectioned, and stained. The areas (μm^2^) of individual cells were measured automatically using the KS400 Image Analysis System (Carl Zeiss, Germany) and 1,000 adipocytes per animal were evaluated.

### Apoptosis and proliferation assays

Cell turnover in adipose tissue due to apoptosis was determined by terminal deoxynucleotidyl transferase-mediated dUTP-biotin nick end labeling (TUNEL) assays. The paraffin-embedded VAT sections were analyzed with the *In situ* Apoptosis Detection Kit (MK500, TaKaRa). We used the protocol recommended by the manufacturer. A positive control for the TUNEL assay was made by incubating sections for 15 min at 37 °C with 1 μg/mL DNase I (D4263-1VL, SIGMA) in 50 mM Tris-HCl, pH 7.5, containing 10 mM MgCl_2_ and 1 mg/mL BSA. The cell proliferation by white adipocytes was detected by immunohistochemical staining for Ki67. The paraffin-embedded VAT sections were de-paraffinized and autoclaved at 121 °C for 15 min in 0.1 M citrate buffer, pH 6.0. The sections were then blocked for 30 min with 10% normal goat serum (S-1000, Vector) in 2% BSA/PBS and incubated overnight with anti-Ki67 primary mouse monoclonal antibody (clone MIB-1, M7240, Dako) diluted to 0.8 μg/mL. The slides were washed and then incubated at room temperature for 30 min with biotin-conjugated goat anti-mouse IgG antibody (E0433, Dako) at a dilution of 1:300. Endogenous peroxidase was blocked for 20 min with 0.3% hydrogen peroxide in methanol, and then the slides were washed. The sections were reacted at room temperature for 30 min with HRP-conjugated streptavidin (P0397, Dako) at a dilution of 1:300. The slides were washed and processed using the DAB reaction (D006, Dojindo). The nuclei were counterstained with hematoxylin.

### Quantification of fatty liver formation

Lipid deposition was detected in whole liver fixed overnight in 4% PFA. The fixative was subsequently replaced with increasing concentrations of sucrose (10, 20, and 30% in PBS, for 24 h each). After sucrose replacement, the tissues were frozen-fixed in OCT mounting media in dry ice/acetone and cryosectioned. The sections were stained with Oil Red O and counterstained with hematoxylin. The areas (μm^2^) of lipid deposition per image at the same magnification were measured using ImageJ software (Rasband W; National Institutes of Health, USA).

### Plasma assay

After fasting for 4 h, blood samples were collected from anesthetized mice using BD Microtainer^R^ PST^TM^ Tubes with lithium heparin-Amber (Becton, Dickinson and Company). Plasma were separated by centrifugation at 2,000 × *g* for 20 min at 4 °C and cryopreserved at −80 °C until use. The plasma levels of triglycerides, total cholesterol, CM cholesterol, VLDL cholesterol, LDL cholesterol, and HDL cholesterol were measured by LipoSEARCH (Skylight Biotech Inc., Akita, Japan).

### Whole body consumption analysis: indirect calorimetry measurements

Indirect calorimetry, a noninvasive technique for measuring the mass of oxidation of carbohydrates (CHO) and lipids by analysis of the respiratory gas, was measured using a model ARCO 2000 mass spectrometer (Arco System, Chiba, Japan)^66,67^. Each mouse was caged in an individual metabolic chamber maintained at 24 °C in a 12-h light-dark cycle with ad libitum feeding. The metabolic chamber volume was 220 cm^2^ for the base and 11.5 cm in height. Briefly, room air was pumped through the acrylic metabolic chamber at a rate of 0.4 L/min, and the O_2_ consumption (mL/kg/min) and CO_2_ production (mL/kg/min) were measured every 2.5 min for 24 h. The respiratory exchange ratio, CHO oxidation (mg/kg/min), and fat oxidation (mg/kg/min) were calculated automatically from the O_2_ consumption and CO_2_ production, using the equation of Frayn^68^. These data were based on a 12 h measurement, which was divided into 6 h light/6 h dark periods.

### Insulin and glucose tolerance tests

The insulin tolerance test (ITT) was performed in mice after a 4 h-fasting. Insulin at 0.75 U/kg body weight was injected intraperitoneally. The glucose tolerance test (GTT) was conducted after a 14 h-fasting, and then 2 g/kg body weight of glucose was injected intraperitoneally. After the injection, whole blood was collected from the tail vein at 20 min intervals for ITT and at 15, 30, 60, 90, and 120 min for GTT. The levels of glucose in the whole blood were measured using a ONETOUCH Ultra device (Johnson & Johnson K.K).

### Myosin heavy chain (MHC) isoforms

Myosin heavy chain isoforms were analyzed by electrophoresis, as previously described^69^. Skeletal muscle was frozen in liquid nitrogen, powdered, and then homogenized in 10% SDS buffer (10% SDS; 40 mM dithiothreitol [DTT]; 5 mM EDTA; and 0.1 M Tris-HCl, pH 8.0) supplemented with a protease inhibitor cocktail (Complete Mini, EDTA-free, Roche Diagnostics). After heating at 90 °C for 3 min, the samples were diluted in 2 × sample buffer containing 4% SDS; 100 mM DTT; 0.16 M Tris-HCl, pH 6.8; 43% glycerol; and 0.2% bromophenol blue. The separating gel consisted of 35% glycerol; 8% acrylamide-N,N′-methylenebisacrylamide (Bis-acrylamide; 99:1); 0.2 M Tris-HCl, pH 8.8; 0.1 M glycine; 0.4% SDS; 0.1% ammonium persulfate (APS); and 0.05% N,N,N′,N′-tetramethylethylenediamine (TEMED). The stacking gel consisted of 30% glycerol; 4% bis-acrylamide (50:1); 70 mM Tris-HCl, pH 6.8; 4 mM EDTA; 0.4% SDS; 0.1% APS; and 0.05% TEMED. The lower running buffer consisted of 0.05 M Tris base; 75 mM glycine; and 0.05% SDS. The upper running buffer was 6-fold the concentration of the lower running buffer, and β-ME was added to 0.12%. A 50 ng sample of total protein was electrophoresed per lane. Electrophoresis was performed at 10 mA at 4 °C for 40 min and then at 140 V at 4 °C for 22 h. The gel was stained with a silver staining kit (Silver Stain KANTO III; Kanto Chemicals). The bands of interest were quantified by densitometry using ImageJ software (Rasband W; National Institutes of Health, USA).

### Western blot analysis

Frozen skeletal muscle was powdered under liquid nitrogen and homogenized in ice-cold RIPA buffer containing 5 mM Tris-HCl, pH 7.4; 1% NP-40; 0.1% SDS; 1% sodium deoxycholate; 5 mM EDTA; 150 mM NaCl; protease inhibitor cocktail; and phosphatase inhibitor cocktail (PhosSTOP; Roche Diagnostics). The homogenates were centrifuged at 10,000 × *g* for 15 min at 4 °C. The resulting supernatant was mixed with 3 × sample buffer (Blue Loading Buffer; Cell Signaling Technology, Danvers, MA, USA) and heated at 95 °C for 5 min. Then, a 24 μg sample of total protein was electrophoresed on a polyacrylamide gel (4–12% gradient, NuPAGE® SDS-PAGE Gel system, Thermo Fisher Scientific Inc.) and then transferred to polyvinylidene difluoride (PVDF) membranes. The membranes were stained with Ponceau S (Ponc) and Ponc-stained Images were used to verify equal loading in all lanes^70^. Membranes were then destained with Tris-buffered saline containing 0.1% Tween-20 (TBST) and blocked with 5% skim milk in TBST for 1 h at room temperature. Membranes were then washed and incubated overnight with primary antibodies at 4 °C. The primary antibodies used were: anti-TIM23 antibody (1:1,000 dilution, #611222, BD Biosciences), anti-TOM20 (FL-145) antibody (1:500, sc-11415, Santa Cruz Biotechnology), and anti-PGC1α antibody (1:1,000, ab54481, abcam). After reactions with the primary antibodies, the membranes were washed and incubated with either horseradish peroxidase-conjugated anti-rabbit antibodies or anti-mouse antibodies for 1 h at room temperature. After washing, chemiluminescence reagents (Luminata Forte; Millipore) were used to facilitate detection of the protein bands. Images were scanned with Amersham Imager 600 (GE Healthcare, Tokyo, Japan). Band intensities were measured on the captured images using ImageJ.

### Statistical analysis

Statistical analysis was performed with GraphPad Prism 7 software (San Diego, CA) using unpaired *t* tests, one-way ANOVA, and two-way ANOVA. *P* < 0.05 was considered statistically significant.

## Electronic supplementary material

supplemental manuscript and figures
