# Supplementary material for: Perlecan, a heparan sulfate proteoglycan, regulates systemic metabolism with dynamic changes in adipose tissue and skeletal muscle
Source: Sci Rep. 2018 May 17;8:7766. doi: 10.1038/s41598-018-25635-x (PMC5958100; doi:10.1038/s41598-018-25635-x)
Supplement: Supplementary file 1 — supplemental manuscript and figures [file 41598_2018_25635_MOESM1_ESM.pdf]

Perlecan, a heparan sulfate proteoglycan, regulates systemic metabolism with dynamic changes in adipose tissue and skeletal muscle

Yuri Yamashita[1,2], Satoshi Nakada[3], Toshinori Yoshihara[4], Takeshi Nara[5], Norihiko Furuya[2], Takashi Miida[6], Nobutaka Hattori[2], and Eri Arikawa-Hirasawa[1,2,]*

[1]Aging Biology in Health and Disease, [2]Department of Neurology, and [6]Department of Clinical Laboratory medicine, Juntendo University Graduate School of Medicine, Tokyo 113-8421, Japan. [3]Japanese Center for Research on Women in Sport and [4]Department of Exercise Physiology, Juntendo University Graduate School of Health and Sports Science, Chiba 270-1695, Japan. [5]Faculty of Pharmacy, Iwaki Meisei University, Fukushima 970-8551, Japan.

*Correspondence and requests for materials should be addressed to E.AH.

Eri Arikawa-Hirasawa: at: Juntendo University Faculty of Medicine, Research Institute for Disease of Old Age, Building 10, Room 606, 2-1-1 Hongo, Bunkyo-ku, Tokyo 113-8421, Japan (email: ehirasaw@juntendo.ac.jp)

SUPPLEMENTARY INFORMATION

Supplementary information contains

Supplementary Figure legend & Supplementary Figure 1, 2

Supplementary Figure legend

Supplementary Figure 1: Full length gels and western blot displayed in Figure 5a and 5f.

Supplementary Figure 2: The size of each adipocyte in Figure 2c and 2d was re-plotted (mean ± S.D., n = 3,000-4,000 cells). Data were analyzed by two-way ANOVA with Sidak's multiple comparison. ****p < 0.0001.

Full length gel of MHC isoforms electrophoresis in Figure 5a

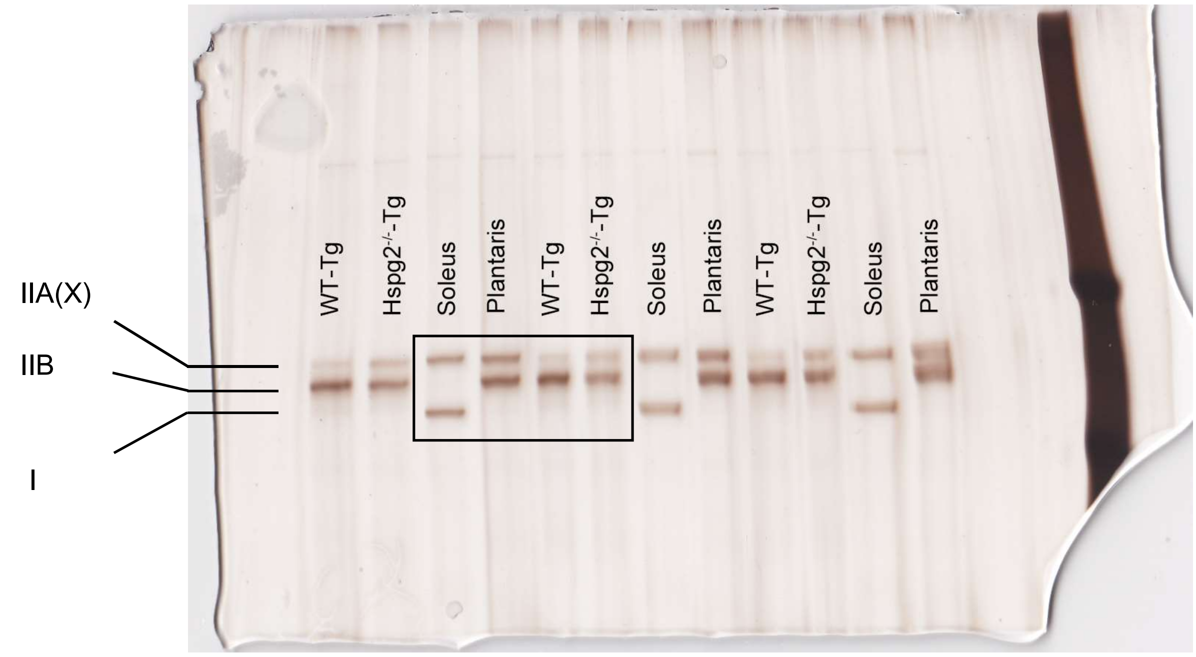

Full length blots in Figure 5f

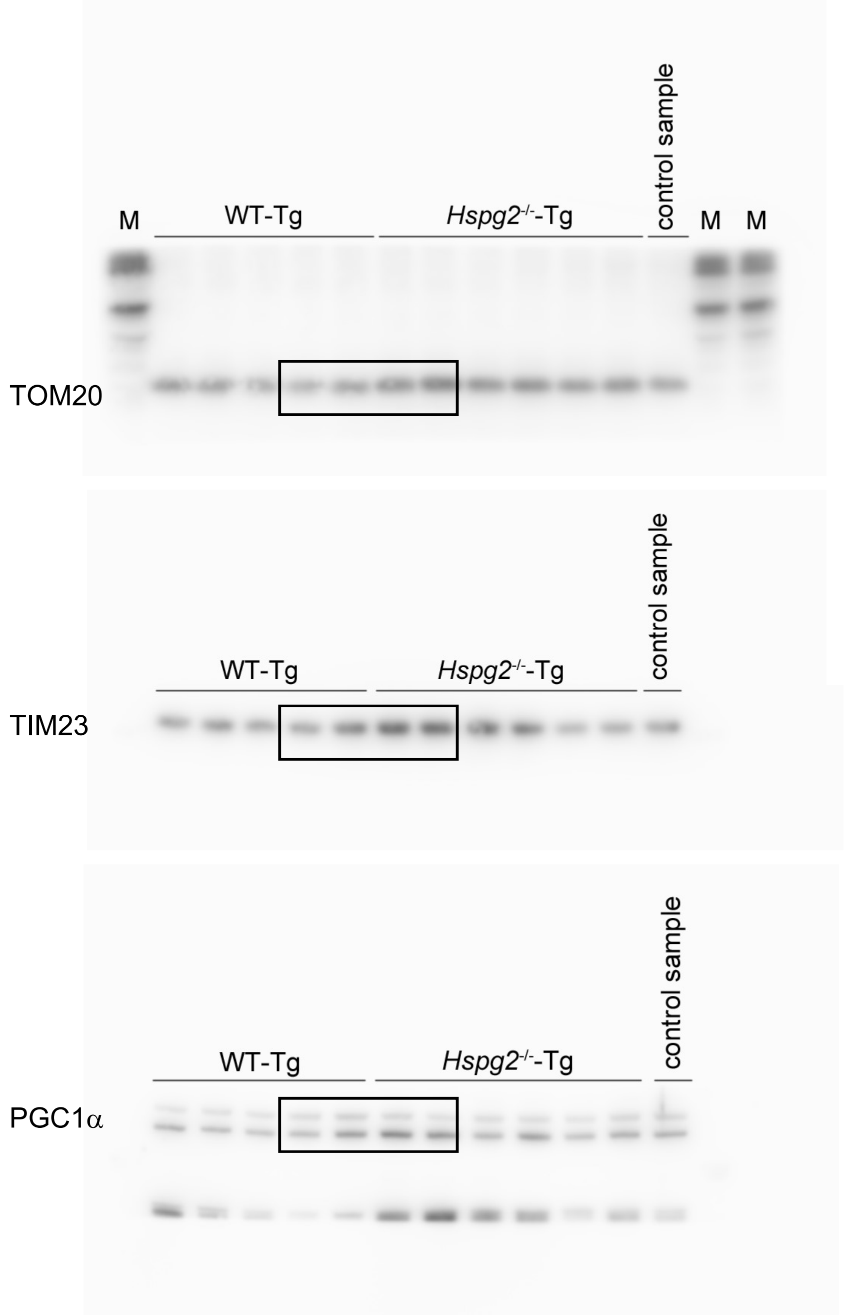

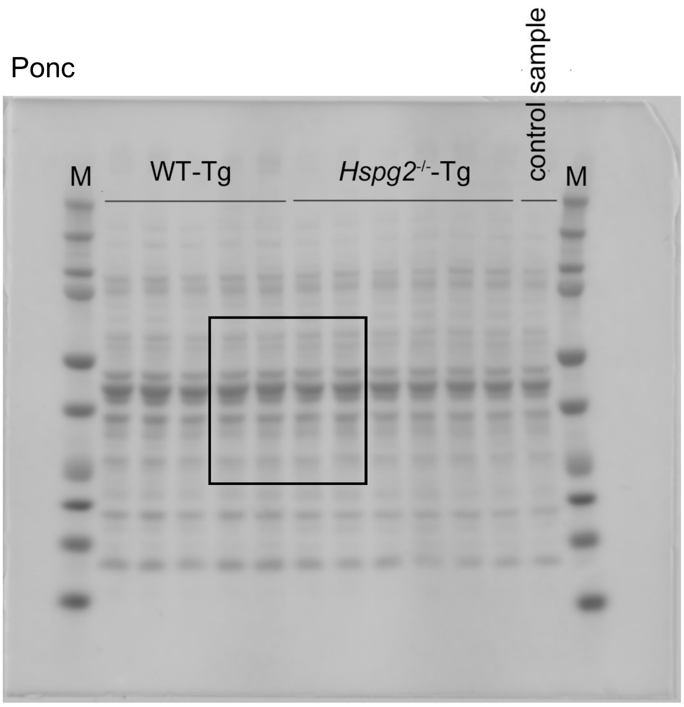

Supplementary Figure-1

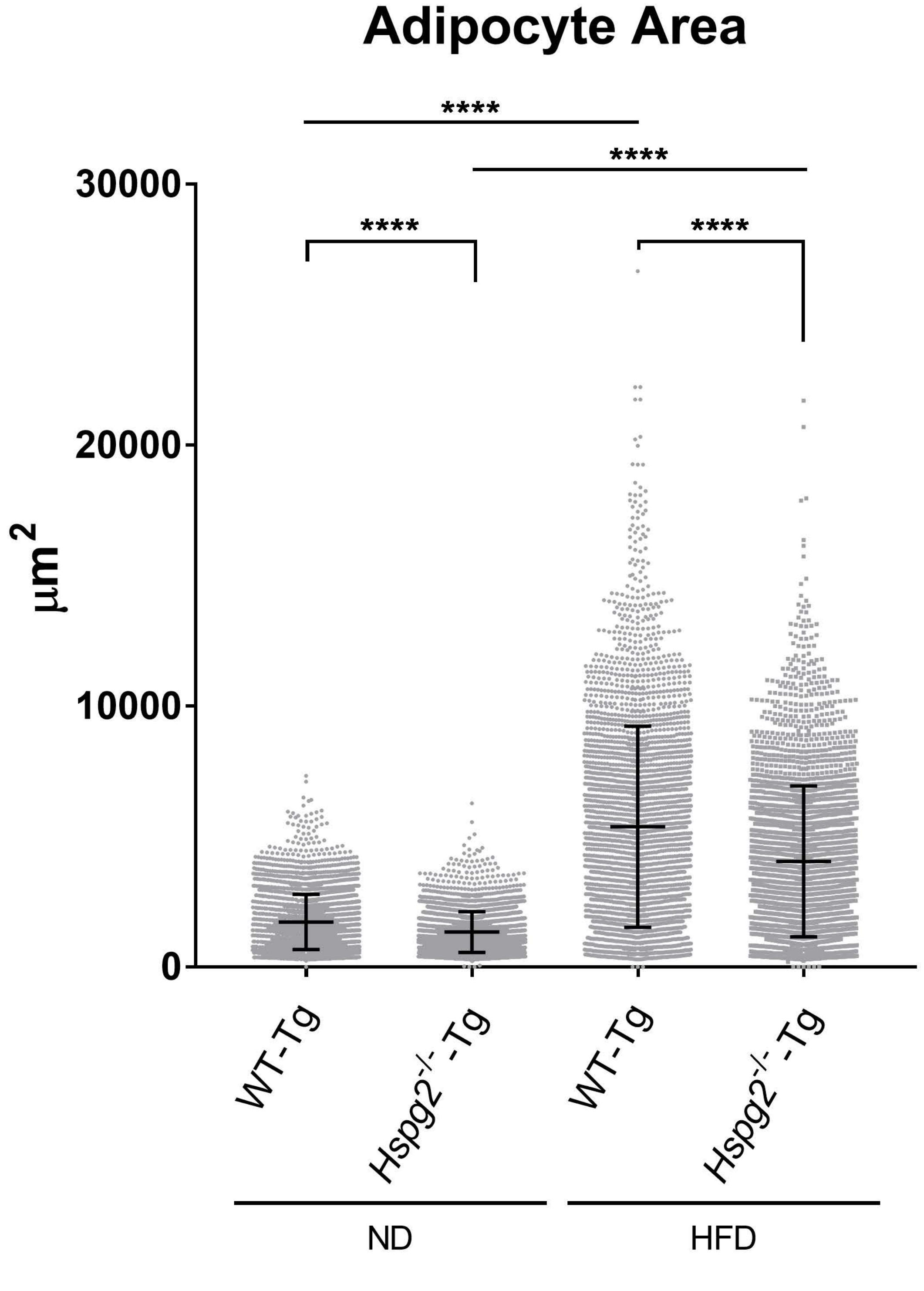

Supplementaly Figure-2
